# OpenDeID Pipeline for Unstructured Electronic Health Record Text Notes Based on Rules and Transformers: Deidentification Algorithm Development and Validation Study

**DOI:** 10.2196/48145

**Published:** 2023-12-06

**Authors:** Jiaxing Liu, Shalini Gupta, Aipeng Chen, Chen-Kai Wang, Pratik Mishra, Hong-Jie Dai, Zoie Shui-Yee Wong, Jitendra Jonnagaddala

**Affiliations:** 1 School of Statistics and Mathematics Zhongnan University of Economics and Law Wuhan China; 2 CGD Health Pty Ltd Canberra Australia; 3 School of Computer Science and Engineering, UNSW Sydney Australia; 4 Department of Computer Science, National Yang Ming Chiao Tung University Hsinchu Taiwan; 5 School of Post-Baccalaureate Medicine, Kaohsiung Medical University Kaohsiung Taiwan; 6 Graduate School of Public Health, St. Luke’s International University Tokyo Japan; 7 The Kirby Institute University of New South Wales Sydney Australia; 8 School of Population Health UNSW Sydney Kensington Australia; 9 NMC Royal Hospital, Khalifa City Abu Dhabi United Arab Emirates

**Keywords:** deidentification, scrubbing, anonymization, surrogate generation, unstructured EHRs, electronic health records, BERT, Bidirectional Encoder Representations from Transformers

## Abstract

**Background:**

Electronic health records (EHRs) in unstructured formats are valuable sources of information for research in both the clinical and biomedical domains. However, before such records can be used for research purposes, sensitive health information (SHI) must be removed in several cases to protect patient privacy. Rule-based and machine learning–based methods have been shown to be effective in deidentification. However, very few studies investigated the combination of transformer-based language models and rules.

**Objective:**

The objective of this study is to develop a hybrid deidentification pipeline for Australian EHR text notes using rules and transformers. The study also aims to investigate the impact of pretrained word embedding and transformer-based language models.

**Methods:**

In this study, we present a hybrid deidentification pipeline called OpenDeID, which is developed using an Australian multicenter EHR-based corpus called OpenDeID Corpus. The OpenDeID corpus consists of 2100 pathology reports with 38,414 SHI entities from 1833 patients. The OpenDeID pipeline incorporates a hybrid approach of associative rules, supervised deep learning, and pretrained language models.

**Results:**

The OpenDeID achieved a best *F*_1_-score of 0.9659 by fine-tuning the Discharge Summary BioBERT model and incorporating various preprocessing and postprocessing rules. The OpenDeID pipeline has been deployed at a large tertiary teaching hospital and has processed over 8000 unstructured EHR text notes in real time.

**Conclusions:**

The OpenDeID pipeline is a hybrid deidentification pipeline to deidentify SHI entities in unstructured EHR text notes. The pipeline has been evaluated on a large multicenter corpus. External validation will be undertaken as part of our future work to evaluate the effectiveness of the OpenDeID pipeline.

## Introduction

### Background and Significance

Electronic health records (EHRs) have become a valuable source for observational research, owing to the accessibility and availability of patient-level data. EHRs contain clinical, imaging, and omics data from patients, enabling clinicians and researchers to generate novel evidence [1]. The EHR data are stored in structured, semistructured, and unstructured formats. Using unstructured data for research purposes is challenging [2]. Unstructured data, including structured or semistructured data, must be deidentified in several scenarios before they can be used for research. Scenarios include where informed consent is not possible to share identified information. As part of the deidentification process, sensitive health information (SHI) must be removed or replaced by surrogates to protect patient privacy [3,4]. The Australian government, similar to the United States with the Health Insurance Portability and Accountability Act (HIPAA) [5], defines several categories of SHI [4]. These include but are not limited to names, dates, any unique identifying numbers or codes, and geographical data, which should be excluded or replaced from EHR text notes prior to secondary use for research purposes. The goal of the deidentification process in unstructured EHR text notes is to identify SHI by inspecting entire medical records. Deidentification by medical experts is time-consuming, error prone, and expensive [6]. In contrast, automated deidentification techniques based on recent advances in artificial intelligence can be used to simplify the entire process [7]. Automated deidentification techniques require an annotated corpus to identify SHI [8].

Over the past 2 decades, several deidentification methods have been developed to identify the SHI in unstructured EHR text notes. Three methods are widely used in the deidentification process, which are rule-based, machine learning–based, and hybrid techniques. Early methods were rule based and required medical professionals to manually develop the rules for most of the time. Researchers from the University of Pittsburgh Medical Center used a rule-based approach to identify and replace SHI while preserving the underlying medical information in pathology reports [9]. Sweeney [10] proposed another rule-based approach for identifying and replacing SHI.MIT-De-id (PhysioNet) software, which is also a rule-based deidentification system developed using nursing progress reports [11]. Zhao et al [12] developed a rule-based model and integrated it into an ensemble framework and achieved the best performance of the model when compared with a non–rule-based model. Similarly, Dehghan et al [13] developed cDeID software to deidentify 7 HIPAA categories. Philter is another deidentification system based on rules incorporating name lists and statistical natural language processing (NLP) to remove SHI from EHR text notes [14].

In recent years, with advancements in artificial intelligence, researchers have proposed traditional machine learning–based approaches and deep learning–based approaches. For example, He et al [15] used conditional random fields (CRFs) with a large number of lexical, orthographic, and dictionary features. In another study, researchers proposed a self-attention mechanism using stacked recurrent neural networks [16]. Contextualized word embeddings and pretrained word embeddings have been examined with bidirectional long short-term memory networks (Bi-LSTM) or Bi-LSTM-CRF in various studies [17-19]. Another recent study proposed an ensemble-based framework to deidentify 5 SHI categories, including person, address, date of birth, identifiers, and phone numbers using Australian hospital discharge summaries [20].

Hybrid approaches have also been proposed to combine rule-based and machine learning–based methods to achieve better performance. For example, Ferrández et al [21] proposed a stepwise hybrid approach, which used CRF, rules, and a dictionary first and then support vector machine. Lee et al [22] presented a hybrid approach using CRF and rules for the automatic deidentification of psychiatric notes. Zhao et al [23] proposed a hybrid approach using recurrent neural networks and text templates. Liu et al [24] developed a hybrid method based on a rules-based method and an ensemble classifier of CRF and Bi-LSTM.

However, there are several limitations to the existing approaches. These methods lack robustness and require comprehensive modifications of rules to achieve better accuracy when applied to EHR text notes data from different health care settings. Many existing deidentification studies have focused either on using rule-based approaches or hybrid approaches based on CRF or Bi-LSTM and rules to identify SHI categories [21-24]. Only a few studies have examined the potential of hybrid approaches that incorporate rules, state-of-the-art pretrained language models, and deep learning [25]. Furthermore, in most of these approaches, the models are trained on a corpus developed from a single center or a corpus prepared from a cohort of patients with a specific disease. As such, there is little evidence on the implementation of these approaches in the real-time processing of EHR text notes deidentification.

### Objective

This study proposes an end-to-end deidentification pipeline called OpenDeID to deidentify real-world Australian unstructured EHR text notes data. The OpenDeID pipeline incorporates a hybrid approach of associative rules, supervised deep learning, and pretrained language models. The best-performing run of OpenDeID was used at a large tertiary teaching hospital in Australia in 2019, deidentifying EHR text notes associated with biobanking in real time. Moreover, the OpenDeID pipeline can also generate realistic surrogates to safeguard SHI [26]. As of 2022, this deployment of the OpenDeID pipeline has processed more than 8000 reports in real time.

## Methods

### OpenDeID Corpus

An OpenDeID corpus was used to develop the OpenDeID pipeline. The OpenDeID corpus comprised 2100 pathology reports from 1833 patients with 38,414 SHI entities. The interannotator agreement, measured using Cohen κ, was 0.9464. The corpus was created using 2 annotators in 3 different experimental settings. Table 1 lists the 8 SHI categories and their corresponding subcategories annotated in the corpus. The corpus was randomly divided into 3 equal sets: training, validation, and test sets based on the reports. The training set was used for the initial training and validation sets to tune the hyperparameters and early stopping. The models were then trained on the combined training and validation sets, followed by evaluation on the test set. The corpus characteristics and corpus development process are described in detail in [27,28]. The OpenDeID corpus was developed using annotation guidelines developed by Stubbs et al [29]. The corpus is available at the OpenDeID-Corpus GitHub repository [30].

**Table 1:** SHI^a^ categories and subcategories for deidentification annotation.

SHI category	SHI subcategory
Name	PATIENT, DOCTOR, USERNAME
Profession	No subcategories
Location	HOSPITAL, ORGANIZATION, STREET, CITY, STATE, COUNTRY, ZIP CODE, OTHER
Age	No subcategories
Date	No subcategories
Contact	PHONE, FAX, EMAIL, URL, IP ADDRESS
ID	SSN, MEDICAL RECORD, HEALTH PLAN, ACCOUNT, LICENSE, VEHICLE, DEVICE, BIOID, IDNUM
Other	No subcategories

^a^SHI: sensitive health information.

### OpenDeID

#### Overview

**Figure 1:**
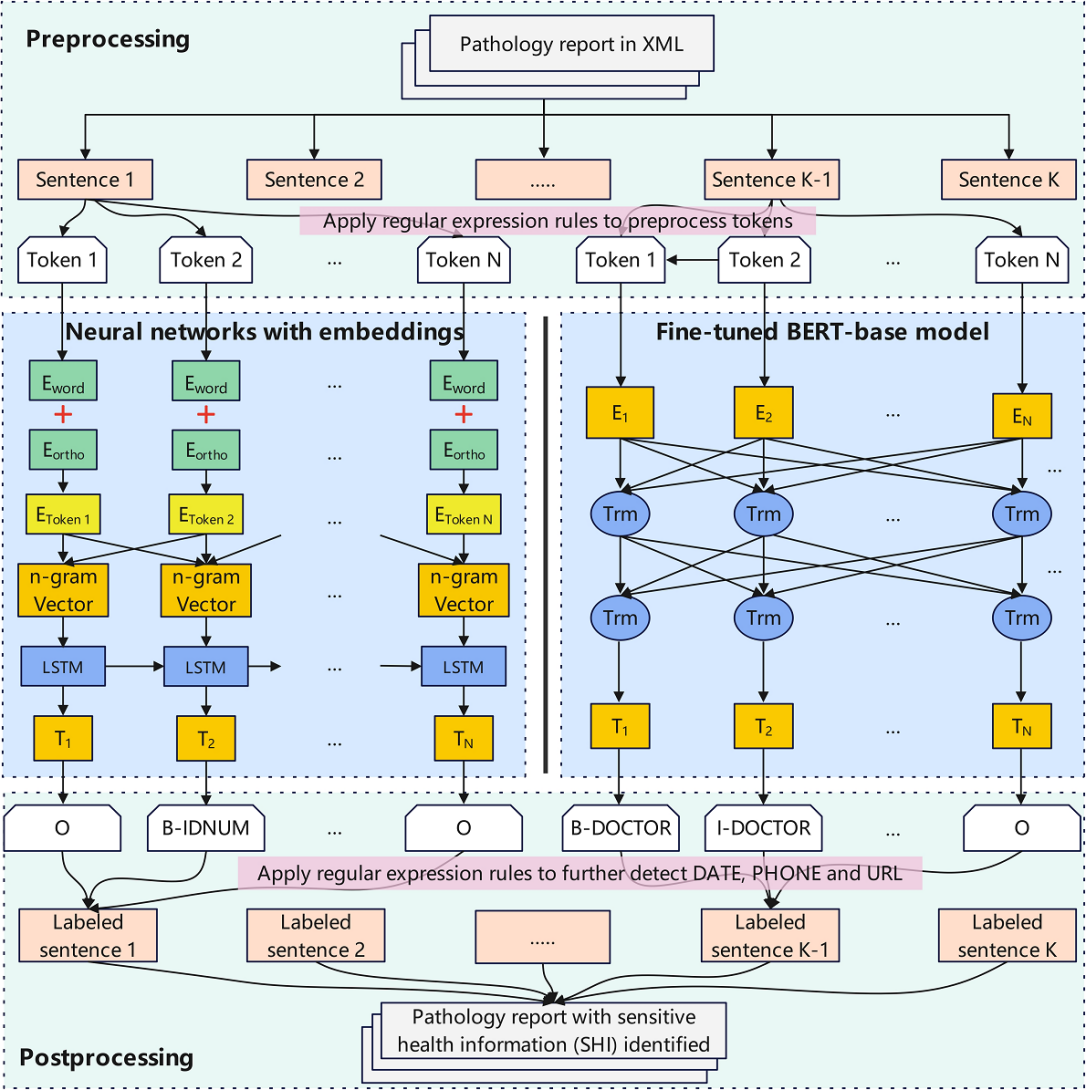
Conceptual overview of the pipeline of OpenDeID. BERT: Bidirectional Encoder Representations from Transformers; LSTM: long short-term memory.

OpenDeID uses a step-by-step pipeline approach for deidentifying sensitive information. The first step is preprocessing, where pathology reports in the XML format are cleaned using NLP methods such as regular expression rules, sentence segmentation, and tokenization. The next step is to build deidentification models. We used two methods: (1) training a neural network model with word embeddings and (2) fine-tuning pretrained Bidirectional Encoder Representations from Transformers (BERT)–base models using transfer learning. The methods were compared and evaluated in terms of their accuracy and performance. The deidentified reports were further processed to generate an output with the appropriate SHI entity tags. Figure 1 shows a conceptual overview of the OpenDeID pipeline. The OpenDeID pipeline is available at the OpenDeID-Pipeline GitHub repository [31]. The main steps are elaborated in the following subsections.

#### Preprocessing

The pathology reports were segmented and assigned tokens using the *Spacy* toolkit. We observed that some of the tokens were incorrectly cascaded, which would impact the SHI tagging. For example, the tokens “PsychiatryChief” and “JMH.Does” should have been “Psychiatry Chief” and “JMH. Does,” respectively. For example, consider “Age: \n\n39Sex”—where just the number “39” should be labeled as an SHI, not “Age” or the entire value “39Sex.” We applied regular expressions–based rules after tokenization to further split and fix the incorrectly cascaded tokens to overcome this problem. The fixed tokens were then updated in the sentences. This is the most common problem in tokenizing, where custom rules need to be added as per the task [32]. We designed the deidentification process as a sentence-tagging task and predicted the labels assigned to tokens in each sentence. These labels were used to indicate different categories of SHI. Our study explored 2 types of tagging schemes: BIO and BIESO [33]. We finally used the BIESO tagging scheme in which “B,” “I,” and “E” indicate that the corresponding token is the “beginning,” “inside,” and “ending” for a certain SHI. “S” indicates that the current token is an SHI consisting of only 1 word. Additionally, “O” refers to the “outside” label, which indicates that the token is not an SHI. Furthermore, we observed that the cascaded tokens were not correctly identified. For example, 2 ID entities were recorded as 1 ID. Subsequently, we tailored the associated cascaded rules for preprocessing to improve the deidentification performance for Australian pathology reports.

#### Experimental Setup

##### Neural Networks With Word Embeddings

Word embedding or word vector is a method where individual words are represented as a vector in a predefined space. It is used for dimensionality reduction and capture word semantics, which can be later used as an input in machine learning models for training and inference. This is required because neural networks only recognize the numerical inputs [34]. In this experimental setting, sentences were further split into tokens and n-grams. The pretrained word embeddings of the word token and its surrounding tokens were used to represent each token. In this experiment, we investigated GloVe (global vectors for word representation), PMC, and Word2vec-OpenDeID corpus and GloVe+PMC+word2vec-OpenDeID corpus embeddings. GloVe is pretrained on Wikipedia and English Giga-word fifth edition [35]. PMC is pretrained on PubMed, PMC texts, and Wikipedia [36]. Word2vec-OpenDeID corpus is pretrained on the OpenDeID corpus with the word2vec algorithm [37]. In the GloVe+PMC+word2vec-OpenDeID corpus, each token was represented by using a concatenation of the above 3 pretrained embeddings. Furthermore, we manually engineered orthographical features [38] based on regular expressions to detect writing patterns of different SHI categories. The manually developed features were encoded using a 1-hot representation and concatenated with the token representation. After activation by a rectified linear unit, the activated representation vectors were fed into a neural network based on long short-term memory (LSTM) to learn the tagging sequence. The network architecture was implemented using Keras software (Google). The minibatch gradient descent along with Adam was used to optimize the parameters. The epoch was set to 20, with a batch size of 8192.

##### Fine-Tuning Pretrained BERT-Base Model

BERT is a machine learning framework designed for NLP tasks. BERT is based on a deep learning model called transformers. The mechanism of transformers includes an encoder that reads and processes the text input and a decoder that produces an output based on prediction for the given input. Previously, language models could read text inputs in a sequential order. BERT, on the other hand, has a bidirectional capability introduced by transformers. There are 2 types of BERT framework: BERT-base and BERT-large. The main difference between them is the number of encoder layers.

BERT is designed to pretrain deep bidirectional representations from unlabeled text data by joint conditioning on both the left and right context in all layers. The fine-tuning step is done by including an additional output layer for deep learning tasks. BERT is evaluated on 11 NLP tasks. Fine-tuning the pretrained BERT model allows it to be used for various downstream tasks with minimal architectural modifications. Semantic and syntactic knowledge in a pretrained large corpus can be transferred to downstream tasks [39]. To achieve the deidentification task, a pretrained BERT-base model was first incorporated with an additional output layer to perform token-level classification, and the model was then fine-tuned on labeled data. In this set of experiments, we examined three contextual clinical and biomedical BERT embeddings: (1) BioBERT: pretrained a BERT model on PubMed abstracts and PubMed Central full-text articles [40], (2) Clinical BioBERT: initialized from BioBERT and pretrained on approximately 2 million notes in the MIMIC-III (Medical Information Mart for Intensive Care) v1.4 database [41], and (3) Discharge Summary BioBERT: initialized from BioBERT and pretrained on the discharge summaries only in the MIMIC-III v1.4 database [41]. Alsentzer et al [41] released clinically trained BERT models fine-tuned atop BioBERT. The BERT-base models were implemented using PyTorch (version 1.10.1; PyTorch) in Python 3.9 (Python Software Foundation). The parameters were optimized using the AdamW Optimizer with a learning rate of 2 × 10^–5^. The batch size was 32 for training and 64 for validation and testing, respectively. The early stopping criterion was used to prevent overfitting. The training was stopped when the token-level accuracy or microaveraged *F*_1_-score in the validation set no longer increased for a certain number of patience epochs. The number of training epochs was 20, with a patience of 5. The sequence length of the input to the BERT-base model was 128. Sentences longer than 128 in length were divided into segments. The default hyperparameters were not fine-tuned for efficient training.

##### Postprocessing

The output from the trained models was postprocessed to produce a report in the XML format with the identified SHI entities. The predicted BIESO tags in the sentences were matched with the original text and combined to assign a corresponding SHI categorical output. Furthermore, regular expression rules using the Python *re* library were applied to detect “DATE,” “PHONE,” and “URL” to further improve the accuracy of the identified SHI categories. For example, in the “DATE” field, we used regular expression rules to find mentions matching the following formats “1/12/2000,” “1-12-2000,” “Jan/12/2000,” “12/Jan/2000,” “Jan-12-2000,” and “12-Jan-2000.” For “PHONE,” we used regular expression rules to find any string with the following format: “1234567890,” “123.456.7890,” and “123-456-7890” to identify 10-digit phone numbers.

An XML file was generated, with the text and SHI annotations for each discharge summary report as the final output. Additionally, if required, the OpenDeID pipeline can generate surrogates. In this study, we have not generated or evaluated surrogates. However, we have incorporated our previous work on surrogates into the pipeline to allow users to generate surrogates [26]. Surrogates can be generated on 6 SHI categories such as name, age, contact, location, date, and ID. For example, the surrogate for a name can be generated by an alphabet shift of fixed length. This is followed by mapping the initial alphabet with another random alphabet and finally selecting a surrogate starting with the new alphabet from the dictionaries prepared. Similarly, a surrogate for a date can be generated by a date shift in the range of 1 to 730 days. For ID and contact surrogates, a custom set of rules can be developed [26].

#### Evaluation

Model performance was evaluated using microaveraged precision, recall, and *F*_1_-scores with strict and relaxed matching [42]. For strict matching, the start and end offsets for the system outputs must exactly match those of the gold standard annotations. Relaxed matching allows tolerance of character offsets. This allows for variations in including “s” and other endings that our system may ignore due to the tokenization. For example, “HM” is identified as a “DOCTOR” tag in gold standard annotations. Strict matching would only take the exact words of the token. Relaxed matching would take tokens such as “HMs” and assign them as “DOCTOR” in the system. The *F*_1_-score is a metric used in machine learning to evaluate the accuracy of a model. It incorporates both the precision and recall scores of the model. The details of how to compute the precision, recall, and *F*_1_-score can be found in Multimedia Appendix 1. The evaluation script is based on the 2016 i2b2 evaluation script [43].

#### Ethical Considerations

This study was approved by the University of New South Wales Sydney Human Research Ethics Committee (HC17749). This research study was undertaken in accordance with the approved ethics application, relevant guidelines, and regulations. Additionally, access to the OpenDeID Corpus was approved by the Secure Research Environment for Digital Health (SREDH) Consortium’s [44] Translational Cancer Bioinformatics working group (SR42-2022).

## Results

### Overview

The corpus contained 2100 pathology reports with 38,414 SHI entities. After preprocessing, 15,85,905 tokens and 144,183 sentences were extracted from the corpus. The length of the sentences ranged from 1 token to 293 tokens, with an average length of 11 tokens. The length of the reports ranged from 106 tokens to 3618 tokens, with an average of 755.19 tokens. The corresponding SHI categories (and subcategories) in the training, validation, and testing data sets are summarized in Table S1 in Multimedia Appendix 1. Six SHI categories and 18 subcategories were identified by using the OpenDeID pipeline. We excluded the “LOCATION: ROOM” and “ID: BIOID” subcategories from the model building when fine-tuning the BERT-base models because these subcategories only appeared once in the entire data set.

### Neural Networks With Different Word Embeddings

Table 2 presents the performance of the LSTM neural network with different word embeddings. The model with a concatenation of all developed embeddings achieved the best microaveraged *F*_1_-scores of 0.9222 in strict matching and 0.9237 in relaxed matching settings. The learning curve for the best-performing model is shown in Figure S1 in Multimedia Appendix 1. We also used 2 strategies to investigate their impact on the overall performance (Table S2 in Multimedia Appendix 1).

**Table 2:** Performance on the test sets using neural networks with different word embedding methods^a^.

Word embeddings	Strict			Relaxed		
	Precision	Recall	*F*_1_-score	Precision	Recall	*F*_1_-score
GloVe^b^+PMC+word2vec-OpenDeID corpus	0.9544	0.8921	0.9222	0.9559	0.8935	0.9237
word2vec-OpenDeID corpus	0.9091	0.7669	0.8320	0.9102	0.7679	0.8330
PMC	0.9550	0.8749	0.9132	0.9565	0.8763	0.9146
GloVe	0.9101	0.7470	0.8206	0.9112	0.7479	0.8215

^a^Precision, recall, and *F*_1_-score are microaveraged measures under relaxed settings.

^b^GloVe: global vectors for word representation.

### Fine-Tuning Different Pretrained BERT-Base Models

The performance of the different biomedical and clinical BERT-base models is shown in Table 3. For the overall performance, the best-performing model was Discharge Summary BioBERT with a microaveraged *F*_1_-score of 0.9374 for strict matching and 0.9401 for relaxed matching. However, the difference between the best- and worst-performing BERT-base models was only 0.0014 for strict matching and 0.0015 for relaxed matching, which can be considered minor. The learning curve for the discharge summary BioBERT is presented in Figure S2 in Multimedia Appendix 1. In terms of precision and recall, we observed that the precision of the models was higher than that of the recalls for both strict and relaxed metrics across different BERT-base models. Approximate randomization tests were used to test for statistical significance between different models, and the *P*-values are provided in Table S3 in Multimedia Appendix 1. All the fine-tuned BERT-base models outperformed the neural network models with word embeddings in terms of overall performance. This is mainly achieved by an improvement in the microaveraged recall from 0.8921 (neural network with GloVe+PMC+word2vec-OpenDeID corpus) to 0.9196 (Discharge Summary BioBERT). To evaluate the robustness of the results and the impact of training size, we evaluated different splits of the training and validation sets for fine-tuning the Discharge Summary BioBERT (details in Table S4 and Figure S3 in Multimedia Appendix 1). The results did not change considerably, and we recorded relaxed microaveraged *F*_1_-scores ranging from 0.9361 to 0.9374.

**Table 3:** Performance on the test sets using different fine-tuned pretrained BERT^a^-base models.

BERT-base models	Strict				Relaxed		
	Precision	Recall	*F*_1_-score	Precision		Recall	*F*_1_-score
BioBERT	0.9529	0.9214	0.9369	0.9558		0.9242	0.9397
Clinical BioBERT	0.9555	0.9172	0.9360	0.9582		0.9198	0.9386
Discharge Summary BioBERT	0.9560	0.9196	0.9374	0.9587		0.9222	0.9401

^a^BERT: Bidirectional Encoder Representations from Transformers.

To account for cascaded tokens with a missing space in real life, additional preprocessing rules to splitting the cascaded tokens are implemented by considering the Discharge Summary BioBERT model with the updated OpenDeID pipeline as an example, which results in a 3.04% strict microaveraged *F*_1_-score improvement from 0.9374 to 0.9659, as shown in Table 4. This indicates that a carefully crafted rule-based approach added to the preprocessing phase to boost the performance of the BERT-base model is essential in real-life deidentification problems.

**Table 4:** Performance on the test sets using cascaded ruled applied into BERT^a^-base models.

Run	Strict				Relaxed		
	Precision	Recall	*F*_1_-score	Precision		Recall	*F*_1_-score
Discharge summary BioBERT run without cascading rules	0.9560	0.9196	0.9374	0.9587		0.9222	0.9401
Discharge summary BioBERT run with additional cascading rules	0.9756	0.9564	0.9659	0.9784		0.9592	0.9687

^a^BERT: Bidirectional Encoder Representations from Transformers.

The best-performing run of the OpenDeID pipeline is currently used at a large tertiary hospital to deidentify EHR text notes associated with biobanking. As of 2022, the deployment of OpenDeID pipelines has processed more than 8000 reports in real time.

## Discussion

### Principal Findings

We investigated various configurations of our pipeline using word embeddings and transformer-based methods in conjunction with cascade associative preprocessing rules to deidentify Australian pathology reports. The proposed OpenDeID pipeline consists of 3 steps: preprocessing, modeling, and postprocessing. In the modeling steps, neural networks with word embeddings and fine-tuned pretrained language models were considered and implemented as options. For neural networks with word embeddings, a combination of different word embeddings (GloVe+PMC+word2vec-OpenDeID corpus) achieved the best microaveraged *F*_1_-scores (strict: 0.9222, relaxed: 0.9237). For fine-tuned pretrained language models, the OpenDeID pipeline using Discharge Summary BioBERT with additional preprocessing rules achieved an accurate performance on the test set, achieving the best microaveraged *F*_1_-scores (strict: 0.9659, relaxed: 0.9687).

We evaluated the performance of our pipeline over 6 categories and 18 subcategories commonly found in Australian EHR text notes [45]. Various annotated corpora are available for deidentification. The 2014 i2b2/UTHealth deidentification corpus contains 1304 clinical notes from 296 patients in the United States [46]. The second was the 2016 CEGS N-GRID corpus, which contains 1000 clinical notes from the United States [29]. Uzuner et al [47] developed a corpus containing 889 hospital discharge summaries. The MIMIC-III database contains deidentified clinical data of patients admitted to the Beth Israel Deaconess Medical Center in Boston, United States. Our initial experiments with these corpora showed low generalizability to Australian EHR text notes. Therefore, we used the OpenDeID corpus for training, which is based on Australian EHR text notes data.

To investigate the effectiveness of popular NLP methods in the modeling steps, we implemented neural networks with word embeddings and pretrained language models. It was shown that fine-tuning pretrained BERT-base models outperformed the neural networks with word embeddings in terms of overall performance. For neural networks, various word embeddings were compared, including combinations of PMC, the word2vec-OpenDeID corpus, and GloVe. The result indicates that the concatenation of all word embeddings performed the best. Among the pretrained word embeddings, PMC performed better than the word2vec-OpenDeID corpus and GloVe. There was a negligible difference in performance between the word2vec-OpenDeID corpus and the GloVe word embeddings.

In terms of pretrained language models, we examined different clinical models, including BioBERT, Clinical BioBERT, and Discharge Summary BioBERT, and their deidentification performances were largely similar in this study. Similar results were reported for the performances of BioBERT, Clinical BioBERT, and Discharge Summary BioBERT when they were evaluated on the standard deidentification tasks of i2b2 2006 and i2b2 2014, as shown in Table S5 in Multimedia Appendix 1 [41]. This may be attributed to the lack of necessary patient-identifying knowledge from the upstream pretrained models to be transferred to the downstream deidentification task. This is because many of these clinically pretrained models, such as the Discharge Summary BioBERT and Clinical BioBERT, were pretrained using the MIMIC-III data set, which contains SHI surrogates instead of real SHI. In addition, Ji et al [48] explained the importance of biomedical entity normalization in enhancing the model’s performance. Similarly, reducing the batch size to 16 or 32, as suggested by Devlin et al [49], can improve the overall results. Our experiments showed that a strict microaveraged *F*_1_-score of 0.9374 could be achieved on the test set using the OpenDeID pipeline with Discharge Summary BioBERT training on only 700 annotated reports. This indicates the potential of the pipeline for implementation in low-resource settings.

### Error Analysis

As shown in Table S6 in Multimedia Appendix 1, we further examined the causes of the prediction mistakes identified through error analysis. This step is crucial because it allows us to customize efficient preprocessing methods to deal with frequently occurring, repeating, and mistakenly cascaded tokens. Using the fine-tuned Discharge Summary BioBERT model, we applied the OpenDeID pipeline to detect SHI for these 50 reports. Of the 940 SHI entities, 874 were correctly identified, whereas 66 were mislabeled or not detected by OpenDeID. Among all the SHI subcategories in the selected reports, “NAME: PATIENT,” “LOCATION: ZIP,” “AGE,” and “ID: MEDICAL RECORD” were accurately identified by the OpenDeID pipeline. There were 3 false positives and 4 false negatives related to incorrect gold standard annotations, which should have been treated as true positives. Two examples are presented in Table S7 in Multimedia Appendix 1.

The cascaded rules we implemented prevented 16 false positives and 31 false negatives caused by incorrectly cascaded tokens. These cascaded tokens can be correctly detected by incorporating cascaded rules into an OpenDeID pipeline with a BERT-base model. For instance, without the cascaded rules, a string such as “12G00123,12N01234” would have been treated as a token together, instead of 2 IDs: IDNUM “12G00123” and “12N01234,” resulting in 1 false positive and 2 false negatives outcome. Similarly, we specified the associated rules to (1) identify a token with a comma or semicolon in the middle and (2) add a space after the comma or semicolon and then split the cascaded tokens. The additional cascaded rules can help improve the precision of tokenization and sentence splitting, improving deidentification accuracy. EHR text notes are written as free text, and misspelling and missing space issues are widely observed in the text. Therefore, performing error analysis and applying rule-based preprocessing steps are important to discover and eliminate potential writing problems. Furthermore, different EHR text notes collected by different hospitals may possess different formats, templates, and structures. A one-size-fits-all approach may not be able to fully realize the potential of the deidentification pipeline across all systems. For external data sets that may incorporate this pipeline in the future, it is suggested to study the resulting errors systematically, and in-house modelers can tailor additional rules to incorporate the data properties retained in each specific model. For a detailed example of such an error investigation, refer to the error analysis in Multimedia Appendix 1.

### Limitations and Future Work

This study had some limitations. The recall of OpenDeID must be further improved. Recall measures the ability to retrieve all SHI from clinical texts, which is essential to the deidentification problem because any unidentified SHI could potentially compromise patient privacy. We may consider different loss functions based on recall when optimizing the parameters in the models. Furthermore, we did not fine-tune the hyperparameters in the BERT-base models, which could potentially improve the performances and should be further investigated. It is important to note that our study provides valuable insights and a foundation for deidentification in the context of Australian pathology reports. We anticipate further research to externally validate our pipeline and evaluate the applicability and performance of our approach across different clinical note types. The proposed pipeline was not examined using an external data set. We also intend to further validate the pipeline using other Australian DeID data sets. Although the annotations in the OpenDeID corpus are replaced with synthetic but realistic SHI, it is still possible that the real data might have variations that are not observed in the synthetic annotations. As such, large-scale external validation using real SHI data is necessary to evaluate the effectiveness of the OpenDeID pipeline. Our future work will include the external validation of the pipeline on corpora from other countries and benchmarking against state-of-the-art deidentification tools like OpenDeID.

### Conclusions

This study presents a hybrid deidentification pipeline called the OpenDeID pipeline. The pipeline was deployed at a large tertiary hospital in 2019 and has processed over 8000 reports in real time. The OpenDeID pipeline is evaluated under various settings, using transformer-based neural networks and different configurations of word embeddings. We used the OpenDeID corpus, which consists of 2100 pathology reports extracted from Australian EHR systems to train and evaluate performance. The models were trained on the training and validation sets, followed by evaluation on the test set. Strict and relaxed matching schemes were used for comparative analysis, and the performance was measured using precision, recall, and microaveraged *F*_1_-score. The OpenDeID deidentification pipeline incorporates LSTM with different settings for word embedding or fine-tuned BERT-base models. The pipeline achieved the best performance using a combination of different word embeddings (GloVe+PMC+word2vec-OpenDeID corpus). The best run is the fine-tuned Discharge Summary BioBERT model with an *F*_1_-score of 0.9659. Our systematic error analysis identified minor annotation errors in the corpus and areas of improvement in the OpenDeID pipeline.

## Appendix

Multimedia Appendix 1 Supplementary methods and results.

## Abbreviations

BERT: Bidirectional Encoder Representations from Transformers
Bi-LSTM: bidirectional long short-term memory network
CRF: conditional random field
EHR: electronic health record
GloVe: global vectors for word representation
HIPAA: Health Insurance Portability and Accountability Act
LSTM: long short-term memory
MIMIC-III: Medical Information Mart for Intensive Care
NLP: natural language processing
SHI: sensitive health information
SREDH: Secure Research Environment for Digital Health

## Data Availability

The data sets generated and analyzed during this study are available from the corresponding author on reasonable request.
