# Supplementary material for: OpenDeID Pipeline for Unstructured Electronic Health Record Text Notes Based on Rules and Transformers: Deidentification Algorithm Development and Validation Study
Source: J Med Internet Res. 2023 Dec 6;25:e48145. doi: 10.2196/48145 (PMC10733816; doi:10.2196/48145)

Multimedia Appendix 1: Supplementary Methods and Results

Methods

**Evaluation metrics**

For each SHI category, the precision, recall, and F1 scores can be calculated by true positives, false negatives and false positives as follows:

$${Precision}_{i}=\frac{{TP}_{i}}{{TP}_{i}+{FP}_{i}}$$

$${Recall}_{i}=\frac{{TP}_{i}}{{TP}_{i}+{FN}_{i}}$$

$${F1}_{i}=\frac{2*{Precision}_{i}*{Recall}_{i}}{{Precision}_{i}+{Recall}_{i}}$$

Overall model performance was evaluated using micro-averaged precision, recall, and F1 scores.

$${Precision}_{micro}=\frac{\sum{TP}_{i}}{\sum{TP}_{i}+\sum{FP}_{i}}$$

$${Recall}_{micro}=\frac{\sum{TP}_{i}}{\sum{TP}_{i}+\sum{FN}_{i}}$$

$${F1}_{micro}=\frac{2*{Precision}_{micro}*{Recall}_{micro}}{{Precision}_{micro}+{Recall}_{micro}}$$

Results

**Table S1.** The distribution of SHI categories and summary statistics in the datasets.

| **SHI category: Subcategory** | **Overall** | **Training and Validation** | **Testing** |
| --- | --- | --- | --- |
| NAME: PATIENT | 2118 | 1413 | 705 |
| NAME: DOCTOR | 9671 | 6419 | 3252 |
| LOCATION: ROOM | 1 | 1 | 0 |
| LOCATION: DEPARTMENT | 1840 | 1202 | 638 |
| LOCATION: HOSPITAL | 1952 | 1284 | 668 |
| LOCATION: ORGANIZATION | 5 | 4 | 1 |
| LOCATION: STREET | 1492 | 988 | 504 |
| LOCATION: CITY | 1566 | 1038 | 528 |
| LOCATION: STATE | 1463 | 966 | 497 |
| LOCATION: COUNTRY | 4 | 2 | 2 |
| LOCATION: ZIP | 1519 | 1010 | 509 |
| LOCATION: OTHER | 19 | 15 | 4 |
| AGE | 141 | 82 | 59 |
| DATE | 7665 | 5067 | 2598 |
| CONTACT: PHONE | 7 | 6 | 1 |
| ID: MEDICALRECORD | 2090 | 1393 | 697 |
| ID: BIOID | 1 | 1 | 0 |
| ID: IDNUM | 6860 | 4561 | 2299 |
| No. of Documents | 2100 | 1400 | 700 |
| No. of Sentences | 144183 | 94862 | 49321 |
| No. of Tokens | 1585905 | 1044055 | 541615 |

**Table S2**. Impact of different training strategies.

We also employed two strategies to investigate their impact on the overall performance. The first method is to remove token sequence with all tokens tagging as ‘O’ in the training set to reduce the size of the training set. The second method is to remove the least appeared 3~7 tags out of all tags in the training set to reduce sparsity. Both strategies were tested with the model with the best performed embedding shown in Table 2. By removing the three least appearing tags from the training set (ID-BIOID, LOCATION-ORGANIZATION, and LOCATION-ROOM), the model achieved better PF-scores.

|  | Strict | | | Relaxed | | |
| --- | --- | --- | --- | --- | --- | --- |
|  | P | R | F | P | R | F |
| Removing all ‘O’ sequences | 0.7469 | 0.8668 | 0.8024 | 0.7483 | 0.8685 | 0.8039 |
| Removing 3 infrequent tags | 0.9674 | 0.8893 | 0.9267 | 0.9689 | 0.8908 | 0.9282 |
| Removing 4 infrequent tags | 0.9504 | 0.8933 | 0.921 | 0.9521 | 0.8948 | 0.9226 |
| Removing 5 infrequent tags | 0.9622 | 0.8928 | 0.9262 | 0.9641 | 0.8946 | 0.9281 |
| Removing 6 infrequent tags | 0.9619 | 0.8935 | 0.9264 | 0.9635 | 0.895 | 0.928 |
| Removing 7 infrequent tags | 0.9581 | 0.8864 | 0.9208 | 0.9597 | 0.8879 | 0.8879 |

**Significance Test**

We used approximate randomization to test for statistical significance between different models. The null hypothesis is 2 models will produce identical scores. The test is performed by gathering, shuffling, reassigning predictions from both systems and evaluating the performance changes in evaluation scores like recall, precision or F1 measure. We ran this test with 50000 shuffles.

**Table S3**: *P-*values in the significance test on the micro-averaged scores between different pretrained BERT-base models.

| **Comparisons** | **Strict** | | | **Relaxed** | | |
| --- | --- | --- | --- | --- | --- | --- |
|  | **P** | **R** | **F1** | **P** | **R** | **F1** |
| BioBERT vs Clinical BioBERT | .023 | .003 | .324 | .032 | .002 | .266 |
| BioBERT vs Discharge Summary BioBERT | .005 | .173 | .551 | .006 | .140 | .664 |
| Discharge Summary BioBERT vs Clinical BioBERT | .004 | .041 | .068 | .628 | .041 | .071 |

**Table S4**. Performance on the test sets using Discharge Summary BioBERT model with different experiment settings.

| **Setting** | **Training and Validation (N=1400)** | | **Strict** | | | **Relaxed** | | |
| --- | --- | --- | --- | --- | --- | --- | --- | --- |
|  | **Training** | **Validation** | **P** | **R** | **F1** | **P** | **R** | **F1** |
| 1 | 700 (50%) | 700 (50%) | 0.9560 | 0.9196 | 0.9374 | 0.9587 | 0.9222 | 0.9401 |
| 2 | 840 (60%) | 560 (40%) | 0.9559 | 0.9182 | 0.9367 | 0.9586 | 0.9208 | 0.9393 |
| 3 | 980 (70%) | 420 (30%) | 0.9560 | 0.9188 | 0.9371 | 0.9588 | 0.9215 | 0.9398 |
| 4 | 1120 (80%) | 280 (20%) | 0.9538 | 0.9191 | 0.9361 | 0.9568 | 0.9219 | 0.9390 |

Notes: In the experiments, there are 1400 reports for training and validation and 700 reports for testing. The testing set is held out for evaluation.

**Table S5.** Fine-tuned pretrained BERT-based models’ Performance on i2b2 2006 and i2b2 2014.

|  | **i2b2 2006** | **i2b2 2014** |
| --- | --- | --- |
| BioBERT | 0.948 | 0.930 |
| Clinical BioBERT | 0.947 | 0.925 |
| Discharge Summary BioBERT | 0.948 | 0.927 |

**Table S6.** Error types by SHI categories in the OpenDeID pipeline.

| **SHI category: Subcategory** | **Instances** | **TP** | **No Prediction (FN)** | **Mislabelled (FP)** |
| --- | --- | --- | --- | --- |
| NAME: PATIENT | 51 | 51 | 0 | 0 |
| NAME: DOCTOR | 228 | 214 | 14 | 6 |
| LOCATION: DEPARTMENT | 48 | 47 | 1 | 1 |
| LOCATION: HOSPITAL | 53 | 48 | 5 | 4 |
| LOCATION: STREET | 41 | 37 | 4 | 0 |
| LOCATION: CITY | 46 | 44 | 2 | 0 |
| LOCATION: STATE | 40 | 39 | 1 | 0 |
| LOCATION: ZIP | 41 | 41 | 0 | 0 |
| AGE | 3 | 3 | 0 | 1 |
| DATE | 181 | 178 | 3 | 6 |
| ID: MEDICALRECORD | 50 | 50 | 0 | 0 |
| ID: IDNUM | 158 | 122 | 36 | 13 |
| **Total** | 940 | 874 | 66 | 31 |

Note: LOCATION: ORGANIZATION, LOCATION: COUNTRY, LOCATION: OTHER, and CONTACT: PHONE entities are not reported in the table because they did not exist in the 50 reports.

**Table S7.** Annotation errors in the OpenDeID corpus.

| **Annotation errors in the OpenDeID corpus** | **Example** |
| --- | --- |
| Case 1:  Text: This case was discussed with Dr D Gordon-Thomson. | Gold Standard: Gordon-Thomso (DOCTOR)  OpenDeID pipeline: Gordon-Thomson (DOCTOR) |
| Case 2:  Text: PaLMS Central Coast SC-12-000370/Reviewed by Professors Scolyer and McCarthy | Gold Standard: C-12-0003701 (ID: IDNUM)  OpenDeID pipeline: SC-12-0003701 (ID: IDNUM) |

**Figure S1**. Training and validation accuracy and loss in each epoch using neural network model with GloVe+PMC+word2vec-OpenDeID corpus embeddings.


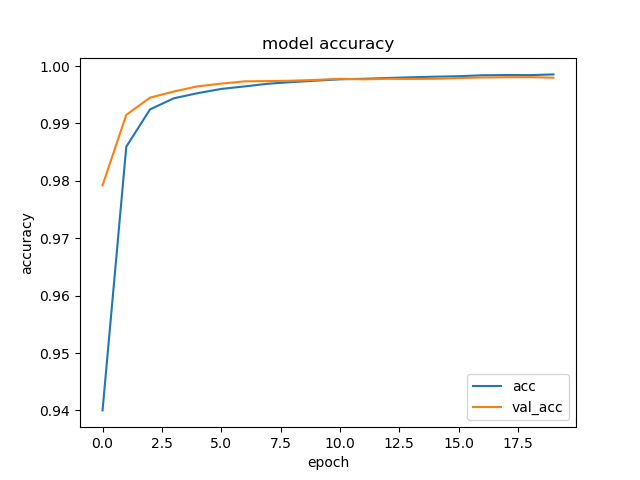

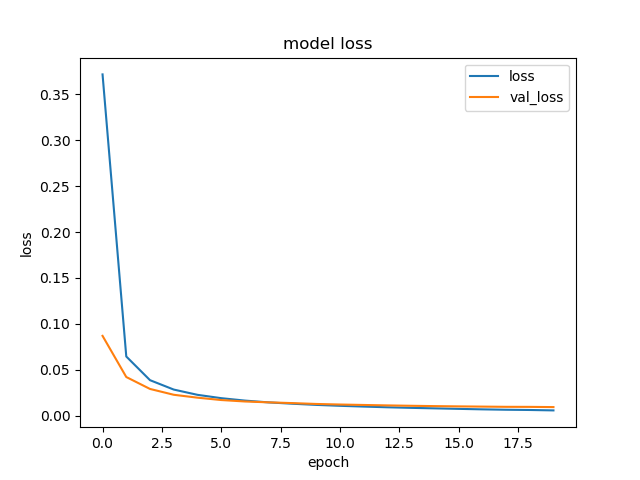


**Figure S2**. Training and validation accuracy and loss in each epoch using Discharge Summary BioBERT.


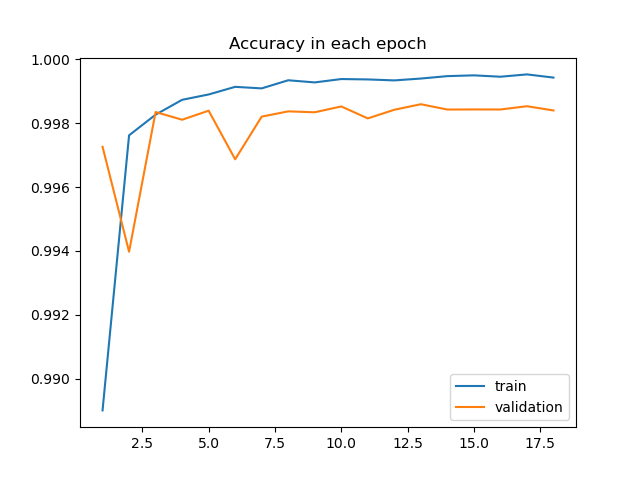

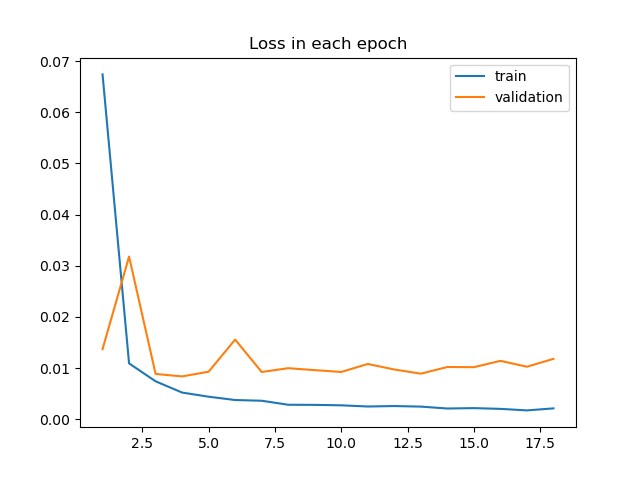


**Figure S3.** Representation of recall with strict and relaxed matching with different experiment settings of training and validation sets in Supplementary Table 2.


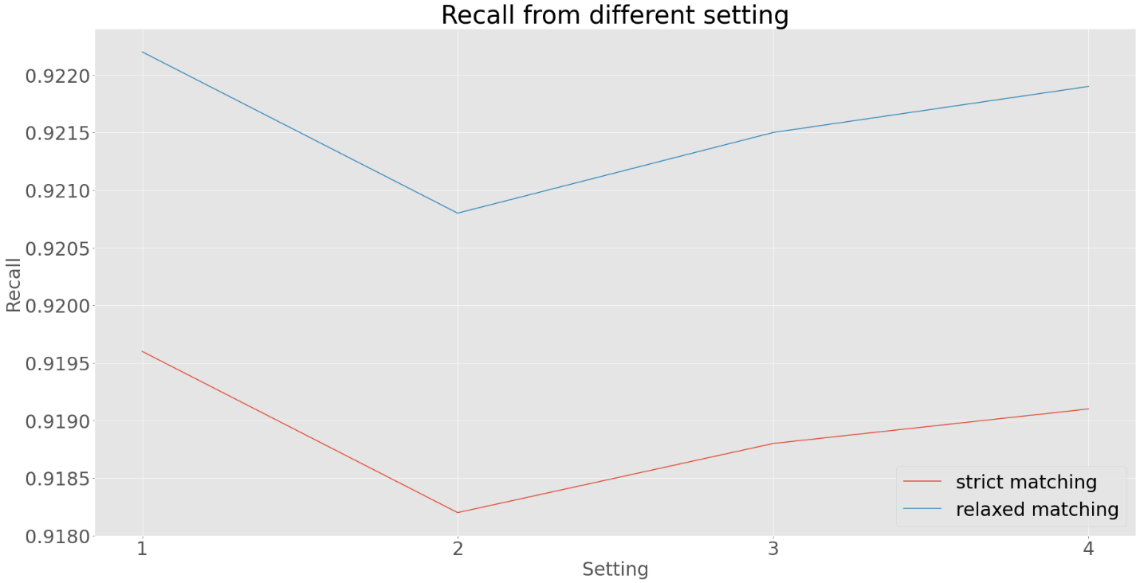

Supplement: Multimedia Appendix 1 [file jmir_v25i1e48145_app1.docx]
